# Exploring Acetyl-CoA-Mediated Beta-Hydroxybutyrate Enrichment in Yeast: A Mechanistic Link to Longevity

**DOI:** 10.1093/geroni/igaf122.2542

**Published:** 2025-12-31

**Authors:** Swarup Mishra, Connor Oakes, Elisa Enriquez Hesles, Jeffrey Smith

**Affiliations:** University of Virginia, Charlottesville, Virginia, United States; University of Virginia, Charlottesville, Virginia, United States; University of Virginia, Charlottesville, Virginia, United States; University of Virginia School of Medicine, Charlottesville, Virginia, United States

## Abstract

Aging predisposes organisms to diseases ranging from diabetes to Alzheimer’s, driving interest in interventions like Caloric Restriction (CR), which extends lifespan across species. While CR’s benefits are well-documented, its metabolic mechanisms remain incompletely understood. This study investigates β-hydroxybutyrate (βHB), a ketone body, in CR-mediated longevity using Saccharomyces cerevisiae. Despite the absence of canonical ketogenesis enzymes (HMG-CoA lyase, OXCT1) in yeast, we detect βHB for the first time in stationary-phase and CR conditions via metabolomics, GC-MS, and luminescence assays. Alternative carbon sources and supplementing yeast media with acetoacetate drive βHB levels. In addition, we identify mitochondrial acetyl-CoA metabolism (ACS1) and mtFAS pathways to be critical for βHB enrichment during CR. βHB supplementation extends chronological lifespan dose-dependently, mirroring CR’s effects, and combined CR + βHB yields additive lifespan extension. Transcriptomic profiling reveals 156 overlapping genes and 268 βHB-exclusive targets, suggesting shared (TCA cycle, amino acid metabolism) and βHB-specific mechanisms (mitochondrial translation, stress response). These findings resolve yeast’s unexpected capacity for ketogenesis and establish it as a model for dissecting βHB’s role in aging. This work also elucidates conserved nodes linking ketogenesis to longevity and positions yeast as a platform for exploring CR-mimicking therapeutics.

